# Feasibility, Reproducibility and Validation of Right Ventricular Volume and Function Assessment Using Three-Dimensional Echocardiography

**DOI:** 10.3390/diagnostics11040699

**Published:** 2021-04-14

**Authors:** Tom De Potter, Caroline Weytjens, Andreea Motoc, Maria Luiza Luchian, Esther Scheirlynck, Bram Roosens, Kaoru Tanaka, Laura Houard, Steven Droogmans, Bernard Cosyns

**Affiliations:** 1 Faculty of Medicine and Pharmacy, Vrije Universiteit Brussel, Laarbeeklaan 103, 1090 Brussels, Belgium; 2 Centrum Voor Hart-en Vaatziekten (CHVZ), Department of Cardiology, UZ Brussel, Laarbeeklaan 101, 1090 Brussels, Belgium; caroline.weytjens@uzbrussel.be (C.W.); andreea.motoc@gmail.com (A.M.); marialuiza.luchian@uzbrussel.be (M.L.L.); esther.scheirlynck@vub.be (E.S.); bram.roosens@uzbrussel.be (B.R.); laura.houard@uzbrussel.be (L.H.); steven.droogmans@uzbrussel.be (S.D.); bernard.cosyns@uzbrussel.be (B.C.); 3 Radiology Department, UZ Brussel, Laarbeeklaan 101, 1090 Brussels, Belgium; kaoru.tanaka@uzbrussel.be

**Keywords:** right ventricle, three-dimensional echocardiography, feasibility, reproducibility, validation

## Abstract

Three-dimensional echocardiography (3DE) is advised for right ventricular (RV) assessment. Data regarding the optimal acquisition settings and optimization are still scarce. We aimed to evaluate the feasibility, reproducibility and validation of 3DE for RV volume and function assessment, using cardiac magnetic resonance (CMR) as gold standard. Thirty healthy volunteers and 36 consecutive patients were prospectively included. CMR was performed in the latter. Standard apical four-chamber view (A4CV), focused A4CV and modified A4CV were used for 3DE RV acquisition. Feasibility (and the effect of changes in settings) was evaluated. Intra and interobserver analyses were performed by three observers (expert vs. novice). RV parameters by echocardiography were compared to CMR. Feasibility of acquisition was 16.7% for A4CV, 80.0% for focused A4CV and 16.7% for modified A4CV. Changes in settings had no significant influence on feasibility and further analysis. Intraobserver variability was good in both expert and novice, interobserver variability was good between experienced observers. Compared to CMR, 3DE volumes were significantly lower with fair to moderate correlation (EDV: 91.1 ± 24.4 mL vs. 144.3 ± 43.0 mL (*p* < 0.001), r = 0.653 and ESV: 48.1 ± 16.4 mL vs. 60.4 ± 21.2 mL (*p* < 0.001), r = 0.530, by multi-beat 3DE and CMR respectively). These findings suggest that standardization is needed in order to implement this technique in clinical practice, thus further studies are required.

## 1. Introduction

The prognostic value of right ventricular (RV) function has been evaluated in various conditions, including pulmonary artery hypertension (PAH), ischemic heart disease and heart failure with reduced and preserved ejection fraction [1,2,3,4,5,6]. Moreover, a recent report focused on COVID-19 patients showed that right ventricular dysfunction is independently associated with mortality [7]. Most common two-dimensional echocardiographic (2DE) parameters for the assessment of the RV, such as tricuspid annular plane systolic excursion (TAPSE), peak systolic velocity (s’) and fractional area change (FAC) are relatively easy and reproducible, but provide an indirect measure of RV function [8,9,10]. The accuracy and reliability of 2DE measurements are limited due to the crescentic shape, complex geometry, and anterior position of the RV [11]. Three-dimensional transthoracic echocardiography (3DE) can overcome these limitations, by creating full-volume datasets from which RV volumes and ejection fraction (EF) measurements can be directly extracted [12]. Echocardiography is highly available and implies lower costs. However, cardiac magnetic resonance imaging (CMR) remains the gold standard for RV evaluation [13,14]. The current data on the validation of RV assessment by 3D echocardiography is insufficient [15,16]. Additionally, the impact of acquisition settings and optimization on data analysis has not been extensively studied. Therefore, we aimed to evaluate the feasibility, reproducibility and validation of 3DE for assessing RV volumes and function, using cardiac magnetic resonance (CMR) as gold standard.

## 2. Materials and Methods

**Figure 1:**
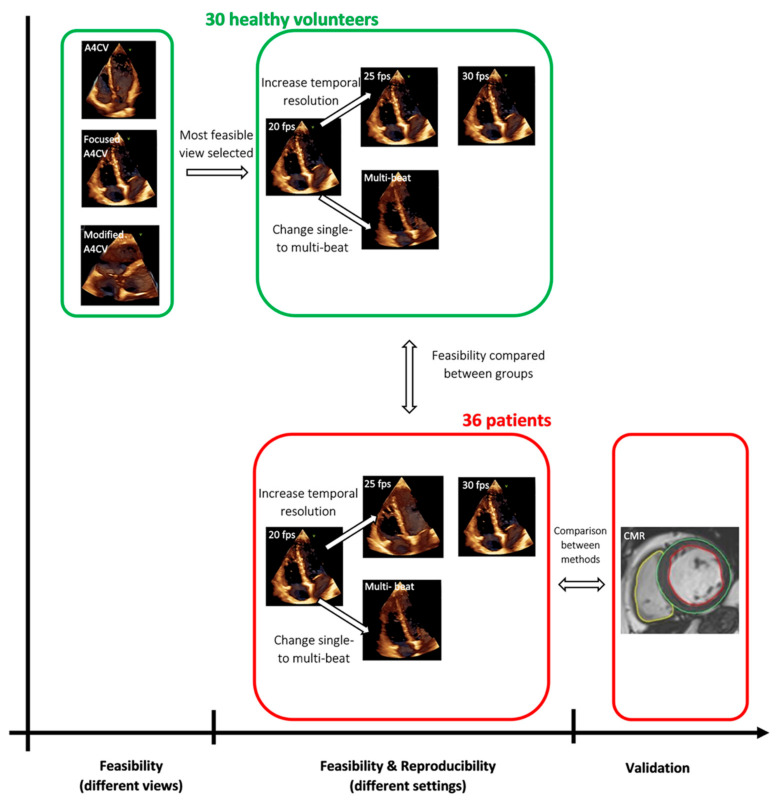
Schematic representation of the study design. (CMR image was reproduced Mikami et al. [17])**.**

A schematic representation of the study design is shown in Figure 1.

### 2.1. Study Population

Sixty-six individuals, divided into two groups, were prospectively included. In the first part of the study, thirty healthy volunteers (28.2 ± 10.7 years, 63.3% male) were recruited between September 2019 and March 2020. Criteria for exclusion were: age < 18 years; history or symptoms of cardiovascular or pulmonary disease (e.g., dyspnea, thoracic pain, palpitations); ongoing or previously use of vasoactive therapy (e.g., anti-hypertensive drugs) or chemo- or radiotherapy, presence of at least one of the following cardiovascular risk factors: (ex-)smoking, hypercholesterolemia, arterial hypertension, diabetes mellitus, dyslipidemia; current pregnancy; professional sports activity; inability to understand the protocol or to give written informed consent. These criteria were checked through a short questionnaire, an electrocardiogram (ECG) and a brief physical examination (blood pressure, heart and lung auscultation).

In the second part of the study, thirty-six consecutive patients (53.3 ± 16.4 years, 61.1% male) with a clinical indication for CMR were enrolled between June 2020 and September 2020. CMR and transthoracic echocardiography (TTE) examination were performed within one hour. Exclusion criteria were: age < 18 years, presence of an intracorporal cardiovascular device (e.g., pacemaker, implantable cardioverter-defibrillator), atrial fibrillation during the examination. Clinical and demographic data were assembled in an electronic case report form (Redcap 7.2.1). The study was carried out according to the ethical principles for medical research involving human subjects, established by Helsinki’s Declaration, protecting the privacy of all participants and the confidentiality of their personal information. All patients provided written informed consent.

### 2.2. Transthoracic Echocardiography

A comprehensive transthoracic echocardiography (TTE) using GE Vivid E95 ultrasound system (GE Vingmed, Horten, Norway) was conducted in all individuals by an experienced echocardiographer (A.M). All patients were in sinus rhythm at the moment of the echocardiography. In addition, full-volume 3D echocardiographic RV datasets were acquired. Sequential single-beat acquisition was performed as follows: a standard apical four-chamber view (A4CV), focused A4CV and modified A4CV with a temporal resolution of 20 frames per second (fps). Focused A4CV was obtained from the standard A4CV by moving the transducer laterally and subsequently tilting it anteriorly. Modified A4CV was obtained from the standard A4CV, by moving the transducer medially followed by tilting it cranially and anteriorly [11]. The feasibility of acquisition of each dataset was assessed in the first group. Visibility of at least two-thirds of the endocardial wall was defined as feasible. Subsequently, single-beat acquisition with a temporal resolution of 25 and 30 fps, and multi-beat acquisition (using 4 to 6 consecutive heartbeats) was performed for the most feasible view. Temporal resolution in single-beat acquisition was increased by reducing the sector width.

3DE RV analysis was performed using GE Healthcare’s 4D Auto RVQ tool of EchoPAC (version 20.3). To reconstruct a 3D RV dataset, a vertical axis was placed manually for alignment through the tricuspid valve (TV) center point and the RV apex. The tricuspid annulus and the RV apex had to be manually indicated. The result of the endocardial border determination was displayed in an end-diastolic/end-systolic multislice 3 × 3 interface, where manual corrections could be performed. This multislice model was checked in all datasets during data analysis to ensure the highest accuracy of endocardial contour tracing at all levels of the right ventricle. Manual corrections were performed if necessary. A time-volume curve and various analytical values of RV volume and function were displayed [18]. End-diastolic and end-systolic volumes were automatically detected by the software.

### 2.3. Reproducibility Analysis

In healthy volunteers (group 1), a novice observer (T.D.P.) performed the quantification of RV volumes and EF. Measurements were repeated after two weeks to provide an intraobserver analysis using the same acquired images. For the interobserver analysis, the measurements were done by an experienced observer (A.M.). In patients (group 2), the second observer (A.M.) did the measurements twice within two weeks to set up an intraobserver analysis. A third medium-experienced investigator (M.L.L.) repeated the measurements for interobserver analysis. During all repeated measurements, observers were blinded from previous results.

### 2.4. Cardiac Magnetic Resonance

CMR imaging was performed using a 1.5 T scanner (MAGNETOM Avanto, Siemens Medical Systems, Erlangen, Germany) with a phased-array cardiac coil. Steady-state free precession dynamic gradient-echo cine loops were acquired using retrospective electrocardiography, during 10- to 15-s breath-holds with the following general parameters: 8-mm slice thickness of the imaging planes, 2.9 ms repetition time; 1.5 ms echo time; 60 degree flip angle; 30–40 ms temporal resolution.

CMR images were analyzed using a commercial dedicated software (cvi42 4.1.8, Circle Cardiovascular Imaging Inc., Calgary, AB, Canada). RV endocardial contours were traced on all short-axis slices of end-diastolic and end-systolic phases detected by an experienced CMR analyzer (K.T.), who was blinded from 3DE measurements. The disk summation method was used for the calculation of the RV end-diastolic and the end-systolic volume. RV ejection fraction was calculated by the standard formula.

### 2.5. Statistical Analysis

Statistical analysis was performed using SPSS version 26.0 (IBM Corporation, Armonk, NY, USA). Normal distribution of continuous quantitative variables was assessed by Kolmogorov–Smirnov tests. Normally distributed continuous data were expressed as mean ± standard deviation (SD), or median (interquartiles) for skewed variables. Categorical variables were presented as numbers with percentages. The intraobserver and interobserver agreement was studied using the Bland–Altman method, whereby the mean difference was presented as the bias, and 95% limits of agreement around the bias expressed as the mean difference ± 1.96 SDs. Baseline characteristics were compared between groups using Student *t*-tests and Fisher’s exact tests for continuous and binominal variables, respectively. Intraobserver and interobserver variability were expressed using intraclass correlation coefficients (ICC). The ICC were quantified by the two-way random-effects model with absolute agreement. An ICC < 0.5 was considered “poor”, 0.5 ≤ ICC < 0.75 was considered “moderate”, 0.75 ≤ ICC < 0.90 was considered “good” and an ICC ≥ 0.90 was considered “excellent” [19]. Systematic differences between measurements were evaluated with Student paired *t*-tests (two-tailed) and Mann–Whitney U tests. *p* values <0.05 were considered statistically significant. Pearson’s correlation coefficients (r) were used to assess the relationship between 3DE- and CMR-derived RV volumes and EF. A Pearson’s correlation coefficient was considered “poor” when 0 < r < 0.3; “fair” when 0.3 ≤ r < 0.6; “moderate” when 0.6 ≤ r < 0.8 and “very strong” when r ≥ 0.8 [20].

## 3. Results

### 3.1. Study Population

Baseline demographic characteristics of the 2 studied groups, are summarized in Table 1. Compared to the healthy volunteers, the patients had a significantly higher age, higher BMI and lower heart rate. The main reasons for CMR referral of patients (group 2) were the evaluation of a potential heart failure (*n* = 8), myocarditis (*n* = 6) and hypertrophic cardiomyopathy (*n* = 5). Baseline clinical characteristics of the patients are summarized in Table 2. Compared to healthy volunteers, the patients had significantly more atrial fibrillation (*p* = 0.042), ventricular extrasystole (*p* = 0.011), heart failure (*p* = 0.011), arterial hypertension (*p* = 0.021), obstructive pulmonary disease (*p* = 0.005), coronary artery disease (*p* = 0.011) and dyslipidemia (*p* = 0.005).

Comprehensive standard echocardiographic examinations in healthy volunteers (group 1) were within the limits of normal. Echocardiographic and CMR characteristics of the patients (group 2) are summarized in Table 3.

### 3.2. Feasibility

In healthy volunteers (group 1), feasibility in standard A4CV was 16.7% (*n* = 5), in focused A4CV 80.0% (*n* = 24) and in modified A4CV 16.7% (*n* = 5). Feasibility in focused A4CV, using different settings was: for 25 fps 83.3% (*n* = 25), for 30 fps 80.0% (*n* = 24) and for multi-beat acquisition 80.0% (*n* = 24). In patients (group 2), feasibility in focused A4CV using different settings (20 fps, 25 fps, 30 fps and multi-beat) was the same: 72.2% (*n* = 26). For each observer, RV volumes in both end-diastole and end-systole decreased as the frame rate was increased. This is shown in Table A1 and Table A2 (Appendix A). Volumes in single beat acquisition using 25 fps were most consistent with multi-beat acquisition (EDV: 91.1 ± 26.8 mL vs. 91.1 ± 16.4 mL (*p* = 1.000) respectively, ESV: 48.3 ± 19.9 mL vs. 48.1 ± 16.4 mL (*p* = 0.942), respectively). RV volumes assessed using different settings in single-beat acquisition are compared with multi-beat acquisition in Table A3 (Appendix A).

### 3.3. Reproducibility

Intraobserver variability in both healthy volunteers (T.D.P, novice observer) and patients (A.M. experienced observer) was good to excellent for volumetric analysis and moderate to good for the assessment of ejection fraction. In healthy volunteers, intraobserver ICC in multi-beat acquisition was: for EDV 0.88 (95% CI 0.67–0.95), for ESV 0.92 (95% CI 0.79–0.97), and for EF 0.84 (95% CI 0.62–0.93). In patients, intraobserver ICC in multi-beat acquisition was: for EDV 0.92 (95% CI 0.81–0.96), for ESV 0.88 (95% CI 0.72–0.95), and for EF 0.72 (95% CI 0.38–0.88).

Interobserver analysis in healthy volunteers (T.D.P. vs. A.M., novice vs. experienced observer) was poor to moderate for all the analyzed parameters. Interobserver analysis in patients (A.M. vs. M.L.L, experienced vs. medium-experienced observer) was good to excellent for all the analyzed parameters. In healthy volunteers, interobserver ICC in multi-beat acquisition was: for EDV 0.64 (95% CI −0.22–0.88), for ESV 0.71 (95% CI −0.11–0.90), and for EF 0.60 (95% CI 0.06–0.83). In healthy volunteers, interobserver ICC in multi-beat acquisition was: for EDV 0.96 (95% CI 0.89 to 0.99), for ESV 0.96 (95% CI 0.89–0.99), and for EF 0.81 (95% CI 0.43–0.94). ICC, student paired *t*-test and Bland–Altman analysis of intra- and interobserver analysis, using multi-beat acquisition, is shown in Table 4. The results of the reproducibility analysis, using single beat acquisition with different settings, is summarized in Appendix A (Table A1 and Table A2).

### 3.4. Validation

**Figure 2:**
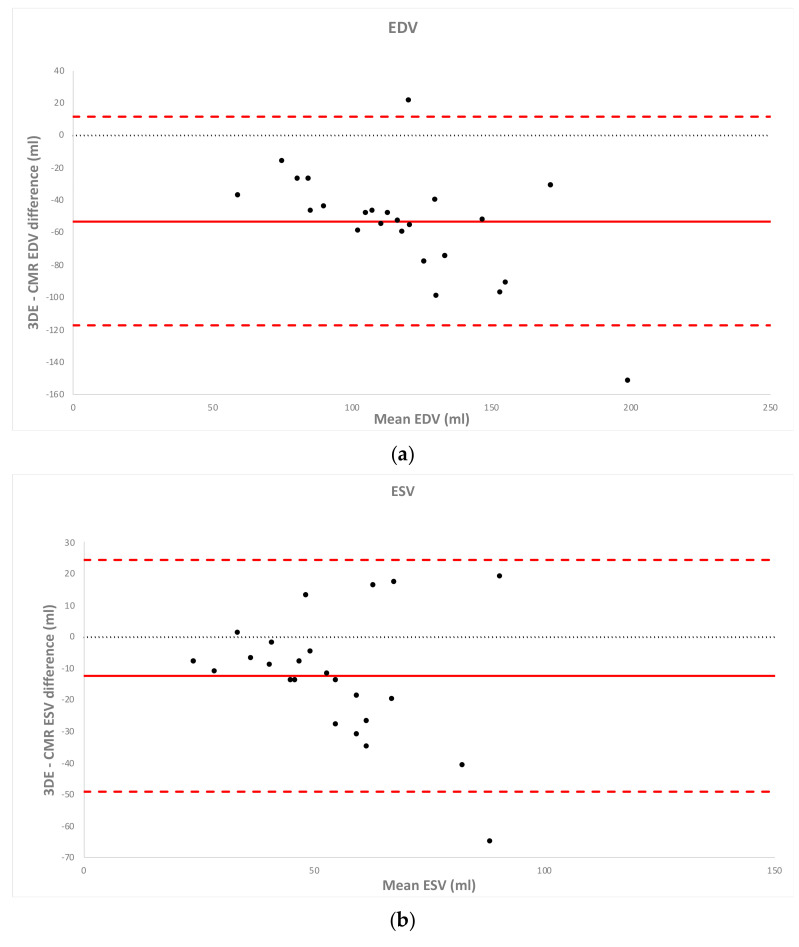

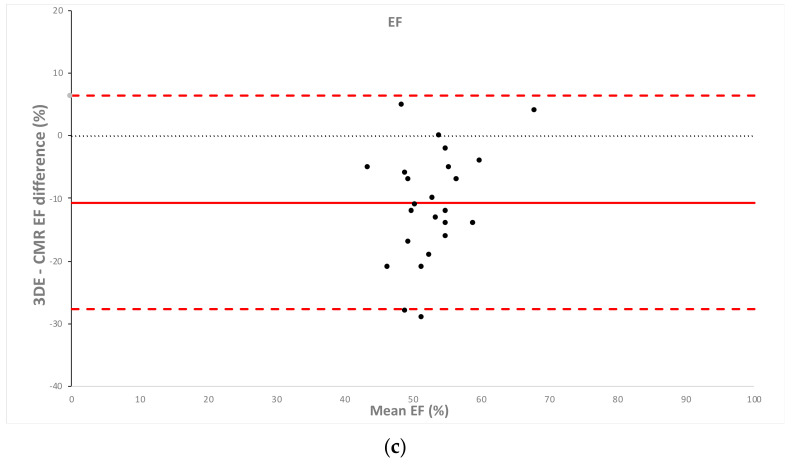
Bland–Altman analysis of bias (red solid line) and 95% limits of agreement (red dashed line) for multi-beat 3DE versus CMR quantification of (**a**) RV EDV, (**b**) RV ESV and (**c**) RV EF in patients. 3DE: three-dimensional echocardiography. CMR: cardiac magnetic resonance. RV EDV: right ventricular end-diastolic volume. RV ESV: right ventricular end-systolic volume. RV EF: right ventricular ejection fraction. LOA: limits of agreement. ICC: intraclass correlation.

RV volumes measured by 3DE were significantly lower than RV volumes measured by CMR (EDV: 91.1 ± 24.4 mL vs. 144.3 ± 43.0 mL (*p* < 0.001) and ESV: 48.1 ± 16.4 mL vs. 60.4 ± 21.2 mL (*p* < 0.001), by multi-beat 3DE and CMR respectively). Pearson’s correlation analysis showed a fair to moderate correlation between volumetric analysis by 3DE and CMR (in multi-beat acquisition: EDV r = 0.653; ESV r = 0.530). Mean volumes measured by 3DE and CMR, paired student *t*-tests, Pearson’s correlation coefficients and Bland–Altman biases and limits of agreement are shown in Table 5. Bland–Altman biases and limits of agreement of 3DE multi-beat acquisition compared to CMR are illustrated in Figure 2.

## 4. Discussion

This study shows that: 1. Feasibility of RV acquisition was the best in focused A4CV. 2. 3DE RV analysis was feasible in both healthy volunteers and patients. 3. Changes in settings and observer experience did not influence the feasibility, nor the reproducibility of RV assessment. 4. Comparison with CMR showed a significant underestimation of RV volumes using 3D echocardiography, but acceptable correlation and agreement between methods.

### 4.1. Feasibility

Similar to previous reports, the present study confirmed that focused A4CV was more feasible for RV acquisition and analysis than standard or modified A4CV [21].

Feasibility was good in both groups, 80.0% in healthy volunteers, and 72.2% in patients. The difference could be explained as the consecutive patients group were substantially older than the healthy volunteers with significantly higher incidence of comorbidities like obesity and COPD, which are known to interfere with image quality [22].

Changing settings, e.g., increasing temporal resolution or changing from single to multi-beat acquisition, had no significant effect on feasibility of acquisition, nor on analysis. This may represent a potential advantage in patients with arrhythmia or an inability to perform a breath-hold, in whom multi-beat acquisition is not possible.

### 4.2. Reproducibility

To the best of our knowledge, this is the first study to investigate the reproducibility of this particular software (4D auto RVQ). The overall reproducibility of RV analysis was comparable to other 3DE software, and other techniques, such as CMR [16,22,23,24]. Reproducibility was good in novice observers. Moreover, a learning curve could be observed, with excellent reproducibility between experienced observers, similar to previous reports [25]. Furthermore, changes in settings did not affect the reproducibility of RV volume analysis. These findings show that 3DE of the RV is a robust technique with low observer dependency, regardless of experience and acquisition settings.

### 4.3. Validation

In line with previous studies [15,23,26], we showed that RV volumes measured by 3DE were significantly lower than those measured by CMR. Although the correlation between RV volumes by 3DE and CMR was acceptable, RV EF by 3DE was poorly correlated to RV EF by CMR. This might be explained by the differences on the volumes’ raw data, which are magnified on the ratio by which EF is calculated [22]. The underestimation of the EDV by 3DE in comparison with CMR is mainly due to a lower spatial resolution leading to less defined endocardial borders. Indeed, CMR tracing is performed wider into the endocardium due to a higher spatial in-plane resolution. In echocardiography, a similar difference in tracing of one millimeter resulted in a volume difference of 11% [27]. Additionally, our study showed a decrease in volume measurement when increasing temporal resolution. This was due to the fact that an increase in temporal resolution leads to a decrease in spatial resolution, which can result in lower volumes [26,27]. Thus, when increasing temporal resolution, the impact of lower spatial resolution is stronger than the benefit of better detection of true end-diastolic and end-systolic momentum. Therefore, the frame rate should not be increased if it is not mandatory.

Multi-beat acquisition combines a high temporal and spatial resolution, but is prone to stitch artifacts in patients with rhythm disturbance and respiratory motion. Improvement in hardware technology ensures that spatiotemporal resolution continuously advances in single-beat acquisition [28]. We observed that volumes measured in single-beat acquisition with an intermediate frame rate (25 fps) corresponded most closely to the multi-beat results. Hence, an intermediate frame rate may guarantee the best trade-off in single-beat acquisition, whereas increasing or decreasing the temporal resolution seems unreasonable.

Additionally, the assessment of RV systolic function by CMR is also prone to bias. The source of errors in evaluation of volumes by CMR Simpson’s method are mainly the demarcation of the basal slice near the atrioventricular valves and outflow tracts, and the large motion of the base through the plane of the short axis (slice thickness was 8 mm), giving rise to significant partial volume effects at the base and apex. This is why the test–retest reproducibility of RV systolic function by CMR is significantly lower than the assessment of left ventricular systolic function [29].

In this study, CMR volumes were calculated from multiple short-axis views. Literature showed that this technique results in higher volumes, compared to when axial images are used [30]. Furthermore, CMR uses an artificial intelligence-based disc summation method. By 3DE, numerous manipulations were needed before semi-automatic 3DE software performed RV reconstruction. Contending 3DE software tools have been adapted to allow automatic tracking and tracing throughout the cardiac cycle, requiring less manual input. Although automated algorithms proved to be accurate and reproducible, sufficient image quality is mandatory [23]. The advancement in transducer and software technology to a fully automated method with higher resolutions can further increase the robustness of 3DE for RV measurements.

The underestimation of volumes with this relatively new software (4D auto RVQ) is comparable to initial versions of similar software, published more than a decade ago [31,32]. However, the feasibility and reproducibility are yet comparable to currently used contending examples. In our opinion, this warrants the use of this software for RV function follow-up via serial assessment. Therefore, we encourage using this software for further studies to eventually introduce it in clinical practice.

### 4.4. Study Limitations

This was a single center study with a limited number of subjects. The age of the healthy volunteers was lower, compared to the patients of the second group. The reproducibility in our study was tested by offline analysis of different observers on the same full-volume datasets. No test–retest analysis was performed. Lastly, it should be noted that no interobserver variability of the CMR analyses was carried out. Larger multicentric studies are warranted to confirm our results.

## 5. Conclusions

This study shows that 3DE acquisition of the right ventricle is feasible in different categories of patients, with no significant impact of changes in settings and that focused A4CV is by far the best imaging view. Reproducibility is good, even in novice observers, and regardless of changes in settings. However, 3DE significantly underestimates RV measurements compared to CMR. These findings suggest that standardization and further studies are needed to implement this technique in clinical practice.

## Figures and Tables

**Table 1:** Baseline clinical and demographic characteristics of the overall study population, with a comparison between healthy volunteers (group 1) and patients (group 2).

Characteristics	Total (*n* = 66)	Healthy Volunteers (*n* = 30)	Patients (*n* = 36)	*p* Value
Demographics				
Age, years	41.9 ± 18.5	28.1 ± 9.5	53.3 ± 16.4	<0.001 *
Male gender (*n*, %)	41 (62.2%)	19 (63.3%)	22 (61.1%)	1.000
Height, cm	175.6 ± 8.5	176.7 ± 8.1	174.7 ± 8.8	0.348
Weight, kg	72.2 ± 13.0	69.3 ± 11.2	74.6 ± 14.0	0.097
BSA, m^2^	1.9 ± 0.2	1.8 ± 0.2	1.9 ± 0.2	0.337
BMI, kg/m^2^	23.3 ± 3.4	22.1 ± 2.3	24.3 ± 3.6	0.007 *
Heart rate, bpm	72.8 ± 10.3	75.8 ± 10.4	70.3 ± 9.7	0.030 *

* statistically significant (*p* < 0.05); BSA: body surface area. BMI: body mass index. bpm: beats per minute.

**Table 2:** Baseline clinical characteristics of the patients (group 2).

Patient Characteristics	(*n* = 36)
Medical history	
Paroxysmal atrial fibrillation (*n*, %)	5 (13.9%)
Ventricular extrasystole (*n*, %)	7 (19.4%)
Heart failure (*n*, %)	7 (19.4%)
Hypertension (*n*, %)	6 (16.7%)
COPD/asthma (*n*, %)	8 (22.2%)
Diabetes mellitus (*n*, %)	4 (11.1%)
CAD (*n*, %)	7 (19.4%)
CVA/TIA (*n*, %)	0 (0.0%)
Dyslipidemia (*n*, %)	8 (22.2%)
Obesity (*n*, %)	4 (11.1%)
Medication	
ACEi or ARB (*n*, %)	7 (19.4%)
Aspirin (*n*, %)	10 (27.8%)
β-blocker (*n*, %)	8 (22.2%)
Calcium channel blocker (*n*, %)	2 (5.6%)
Diuretic (*n*, %)	6 (16.7%)

COPD: chronic obstructive pulmonary disease. CAD: coronary artery disease. CVA: cerebrovascular accident. TIA: transient ischemic attack. PM: pacemaker. ICD: intracardiac defibrillator. ACEi: angiotensin-converting-enzyme inhibitor. ARB: angiotensin receptor blocker.

**Table 3:** Echocardiographic and CMR characteristics of the patients (group 2).

Echocardiography	
Left ventricle	
LV EF (2DE, visually estimated) (%)	53.5 ± 5.8
LV GLS (%)	−16.3 ± 3.7
Diastolic dysfunction (*n*, %)	Total = 12 (33.3%); Type 1 = 11 (30.6%);
	Type 2 = 1 (2.8%)
Left atrium	
LA max volume (2DE, indexed) (mL/m^2^)	26.8 ± 9.9
Right ventricle	
TAPSE (mm)	21.2 ± 0.4
s’ (cm/s)	12.7 ± 2.1
FAC (%)	42.4 ± 11.5
estimated systolic PAP (mmHg)	24.0 ± 6.2
RV EDV (3DE) (mL)	91,1 ± 24,4
RV ESV (3DE) (mL)	48,1 ± 16.4
RV EF (3DE) (%)	47.5 ± 7.4
**CMR**	
RV EDV (mL)	144.3 ± 43.0
RV ESV (mL)	60.4 ± 21.2
RV EF (%)	58.2 ± 5.4

LV EF: left ventricle ejection fraction. LV GLS: left ventricle global longitudinal strain. LA max: left atrium maximum volume. TAPSE: tricuspid annular plane systolic excursion. s’: tricuspid annulus peak systolic velocity. FAC: fractional area change. Estimated PAP: estimated pulmonary arteria pressure. RV EDV: right ventricle end-diastolic volume in multi-beat acquisition. 3DE: three-dimensional echocardiography. RV ESV: right ventricle end-systolic volume in multi-beat acquisition. RV EF: right ventricle ejection fraction in multi-beat acquisition. CMR: cardiac magnetic resonance.

**Table 4:** Results of the reproducibility study of RV parameters in 3DE multi-beat acquisition.

Healthy Volunteers				
Intraobserver	Bias	95% LOA	*p*	ICC, 95% CI
RV EDV (mL)	11.5 ± 19.5	−26.7 to +49.7	0.009 *	0.88 (0.67–0.95)
RV ESV (mL)	5.4 ± 10.3	−14.8 to +25.6	0.018 *	0.92 (0.79–0.97)
RV EF (%)	−0.2 ± 5.1	−10.2 to +9.8	0.841	0.84 (0.62–0.93)
Interobserver	Bias	95% LOA	*p*	ICC, 95% CI
RV EDV (mL)	28.8 ± 21.7	−13.7 to +71.3	<0.001 *	0.64 (−0.22–0.88)
RV ESV (mL)	14.5 ± 12.8	−10.6 to +39.6	<0.001 *	0.71 (−0.11–0.90)
RV EF (%)	1.0 ± 8.1	−14.9 to +16.9	0.558	0.60 (0.06–0.83)
**Patients**				
Intraobserver	Bias	95% LOA	*p*	ICC, 95% CI
RV EDV (mL)	1.6 ± 14.3	−26.5 to +29.7	0.594	0.92 (0.81–0.96)
RV ESV (mL)	−2.0 ± 10.6	−22.8 to +18.8	0.365	0.88 (0.72–0.95)
RV EF (%)	2.1 ± 8.3	−14.2 to +18.4	0.225	0.72 (0.38–0.88)
Interobserver	Bias	95% LOA	*p*	ICC, 95% CI
RV EDV (mL)	1.1 ± 12.1	−22.6 to +56.3	0.729	0.96 (0.89–0.99)
RV ESV (mL)	1.9 ± 7.5	−12.8 to +32.7	0.371	0.96 (0.89–0.99)
RV EF (%)	−1.3 ± 5.5	−12.0 to +29.0	0.410	0.81 (0.43–0.94)

* statistically significant (*p* < 0.05); RV: right ventricle. 3DE: three-dimensional echocardiography. RV EDV: right ventricular end-diastolic volume. RV ESV: right ventricular end-systolic volume. RV EF: right ventricular ejection fraction. LOA: limits of agreement. *p*: paired student *t*-test. ICC: intraclass correlation. CI: confidence interval.

**Table 5:** Results of the validation study. Results of 3DE and CMR analysis of RV parameters are shown, and compared using paired Student *t*-test, Pearson’s correlation coefficient, and Bland–Altman analysis (Bias ± SD and 95% upper LOA—95% lower LOA).

**Single-Beat** **20 fps**	**3DE**	**CMR**	***p***	**r**	**Bias**	**95% LOA**
RV EDV (mL)	99.0 ± 29.1	144.3 ± 43.0	<0.001 *	0.70 * (*p* < 0.001)	−45.3 ± 30.9	−105.9 to +15.3
RV ESV (mL)	51.6 ± 17.0	60.4 ± 21.2	0.019 *	0.57 * (*p* = 0.002)	−8.8 ± 18.1	−44.2 to +26.5
RV EF (%)	48.0 ± 6.2	58.2 ± 5.4	<0.001 *	0.05 (*p* = 0.828)	−10.2 ± 8.0	−26.0 to +5.5
**Single-Beat** **25 fps**	**3DE**	**CMR**	***p***	**r**	**Bias**	**95% LOA**
RV EDV (mL)	91.1 ± 26.8	144.3 ± 43.0	<0.001 *	0.60 * (*p* = 0.001)	−53.2 ± 34.6	−121.0 to +14.5
RV ESV (mL)	48.3 ± 20.0	60.4 ± 21.2	0.006 *	0.49 (*p* = 0.010)	−12.1 ± 20.8	−52.8 to +28.6
RV EF (%)	47.6 ± 10.8	58.2 ± 5.4	<0.001 *	0.12 (*p* = 0.548)	−10.7 ± 11.5	−33.2 to +11.8
**Single-Beat** **30 fps**	**3DE**	**CMR**	***p***	**r**	**Bias**	**95% LOA**
RV EDV (mL)	85.3 ± 26.7	144.3 ± 43.0	<0.001 *	0.64 * (*p* < 0.001)	−59.0 ± 33.2	−124.1 to +6.0
RV ESV (mL)	43.9 ± 17.1	60.4 ± 21.2	<0.001 *	0.52 * (*p* = 0.006)	−16.6 ± 19.1	−53.9 to +20.8
RV EF (%)	49.0 ± 8.9	58.2 ± 5.4	<0.001 *	0.03 (*p* = 0.888)	−9.3 ± 10.3	−29.4 to +10.9
**Multi-Beat** **(4–6 Beats)**	**3DE**	**CMR**	***p***	**r**	**Bias**	**95% LOA**
RV EDV (mL)	91.1 ± 24.4	144.3 ± 43.0	<0.001 *	0.65 * (*p* < 0.001)	−53.2 ± 32.8	−117.5 to +11.1
RV ESV (mL)	48.1 ± 16.4	60.4 ± 21.2	0.003 *	0.53 * (*p* = 0.005)	−12.3 ± 18.7	−49.0 to +24.4
RV EF (%)	47.5 ± 7.4	58.2 ± 5.4	<0.001 *	0.10 (*p* = 0.624)	−10.7 ± 8.7	−27.8 to +6.4

* statistically significant (*p* < 0.05). 3DE: three-dimensional echocardiography. CMR: cardiac magnetic resonance. RV: right ventricle. *p*: paired student *t*-test. r: correlation coefficient. LOA: limits of agreement. RV EDV: right ventricular end-diastolic volume. RV ESV: right ventricular end-systolic volume. RV EF: right ventricular ejection fraction.

## Data Availability

The data presented in this study are available on request from the corresponding author. The data are not publicly available due to the involvement of patient-specific information from hospital-affiliated clinical databases of UZ Brussel.

## Appendix A

**Table A1:** Results of the 3DE reproducibility study in healthy volunteers (group 1). Intra- and interobserver analysis is evaluated using Bland–Altman analysis (Bias ± SD and 95% upper LOA to 95% lower LOA), paired Student *t*-test (*p*) and Intraclass correlation coefficient (ICC).

Intraobserver				
Single-beat 20 fps	Bias	95% LOA	*p*	ICC, 95% CI
RV EDV (mL)	13.2 ± 11.6	−9.5 to +35.9	<0.001 *	0.92 (0.33–0.98)
RV ESV (mL)	9.1 ± 8.0	−6.6 to +24.8	<0.001 *	0.89 (0.22–0.97)
RV EF (%)	−1.9 ± 4.6	−10.9 to +7.1	0.076	0.62 (0.12–0.84)
Single-beat 25 fps	Bias	95% LOA	*p*	ICC, 95% CI
RV EDV (mL)	8.0 ± 20.2	−31.6 to +47.6	0.075	0.90 (0.75– 0.96)
RV ESV (mL)	9.0 ± 14.9	−20.2 to +38.2	0.010 *	0.81 (0.46–0.92)
RV EF (%)	−2.1 ± 5.5	−12.9 to +8.7	0.097	0.72 (0.34–0.88)
Single-beat 30 fps	Bias	95% LOA	*p*	ICC, 95% CI
RV EDV (mL)	10.3 ± 16.1	−21.3 to +41.9	0.008 *	0.92 (0.72–0.97)
RV ESV (mL)	5.5 ± 10.8	−15.7 to +26.7	0.030 *	0.91 (0.76–0.97)
RV EF (%)	−0.1 ± 4.1	−8.1 to +7.9	0.917	0.77 (0.44–0.91)
Multi-beat (4–6 beats)	Bias	95% LOA	*p*	ICC, 95% CI
RV EDV (mL)	11.5 ± 19.5	−26.7 to +49.7	0.009 *	0.88 (0.67–0.95)
RV ESV (mL)	5.4 ± 10.3	−14.8 to +25.6	0.018 *	0.92 (0.79–0.97)
RV EF (%)	−0.2 ± 5.1	−10.2 to +9.8	0.841	0.84 (0.62–0.93)
**Interobserver**				
Single-beat20 fps	Bias	95% LOA	*p*	ICC, 95% CI
RV EDV (mL)	34.5 ± 21.5	−7.6 to +76.6	<0.001 *	0.56 (−0.24–0.86)
RV ESV (mL)	15.6 ± 17.9	−19.5 to +50.7	0.001 *	0.56 (−0.11–0.82)
RV EF (%)	2.7 ± 8.4	−13.8 to +19.2	0.161	0.04 (−1.21–0.60)
Single-beat 25 fps	Bias	95% LOA	*p*	ICC, 95% CI
RV EDV (mL)	37.6 ± 27.9	−17.1 to +92.3	<0.001 *	0.39 (−0.24–0.74)
RV ESV (mL)	17.8 ± 13.6	−8.9 to +44.5	<0.001 *	0.57 (−0.23–0.85)
RV EF (%)	2.7 ± 8.2	−13.4 to +18.8	0.132	0.48 (−0.16–0.78)
Single-beat 30 fps	Bias	95% LOA	*p*	ICC, 95% CI
RV EDV (mL)	31.2 ± 22.7	−13.3 to +75.7	<0.001 *	0.64 (−0.23–0.88)
RV ESV (mL)	12.9 ± 16.8	−20.0 to +45.8	0.002 *	0.67 (0.09–0.87)
RV EF (%)	3.9 ± 7.6	−11.0 to +18.8	0.028 *	0.29 (−0.46–0.69)
Multi-beat (4–6 beats)	Bias	95% LOA	*p*	ICC, 95% CI
RV EDV (mL)	28.8 ± 21.7	−13.7 to +71.3	<0.001 *	0.64 (−0.22–0.88)
RV ESV (mL)	14.5 ± 12.8	−10.6 to +39.6	<0.001 *	0.71 (−0.11–0.90)
RV EF (%)	1.0 ± 8.1	−14.9 to +16.9	0.558	0.60 (0.06–0.83)

* statistically significant (*p* < 0.05). 3DE: three-dimensional echocardiography. RV EDV: right ventricular end-diastolic volume. RV ESV: right ventricular end-systolic volume. RV EF: right ventricular ejection fraction. LOA: limits of agreement. ICC: intraclass correlation.

**Table A2:** Results of the 3DE reproducibility study in patients (group 2). Intra- and interobserver analysis is evaluated using Bland-Altman analysis (Bias ± SD and 95% upper LOA to 95% lower LOA), paired Student *t*-test (*p*) and Intraclass correlation coefficient (ICC).

Intraobserver				
Single-beat 20 fps	Bias	95% LOA	*p*	ICC, 95% CI
RV EDV (mL)	−1.2 ± 15.2	−31.0 to +28.7	0.693	0.92 (0.82–0.96)
RV ESV (mL)	0.6 ± 8.7	−16.4 to +17.6	0.720	0.94 (0.86–0.97)
RV EF (%)	−0.5 ± 5.6	−11.5 to +10.5	0.649	0.85 (0.66–0.93)
Single-beat 25 fps	Bias	95% LOA	*p*	ICC, 95% CI
RV EDV (mL)	0.7 ± 17.1	−32.9 to +34.2	0.847	0.90 (0.78–0.96)
RV ESV (mL)	1.4 ± 11.9	−21.9 to +24.8	0.548	0.92 (0.81–0.96)
RV EF (%)	−1.9 ± 9.5	−20.5 to +16.8	0.326	0.67 (0.27–0.85)
Single-beat 30 fps	Bias	95% LOA	*p*	ICC, 95% CI
RV EDV (mL)	1.1 ± 17.4	−33.1 to +35.3	0.747	0.88 (0.74–0.95)
RV ESV (mL)	−0.3 ± 9.3	−18.5 to +17.8	0.851	0.91 (0.79–0.96)
RV EF (%)	0.3 ± 10.1	−19.4 to +20.0	0.871	0.38 (−0.47–0.72)
Multi-beat (4–6 beats)	Bias	95% LOA	*p*	ICC, 95% CI
RV EDV (mL)	1.6 ± 14.3	−26.5 to +29.7	0.594	0.92 (0.81–0.96)
RV ESV (mL)	−2.0 ± 10.6	−22.8 to +18.8	0.365	0.88 (0.72–0.95)
RV EF (%)	2.1 ± 8.3	−14.2 to +18.4	0.225	0.72 (0.38–0.88)
**Interobserver**				
Single-beat 20 fps	Bias	95% LOA	*p*	ICC, 95% CI
RV EDV (mL)	7.7 ± 20.5	−32.5 to +84.3	0.183	0.88 (0.64–0.96)
RV ESV (mL)	1.6 ± 14.3	−26.4 to +66.1	0.687	0.84 (0.51–0.95)
RV EF (%)	3.0 ± 5.9	−8.6 to +22.7	0.083	0.77 (0.30–0.92)
Single-beat 25 fps	Bias	95% LOA	*p*	ICC, 95% CI
RV EDV (mL)	−4.0 ± 9.0	−21.7 to +51,5	0.121	0.97 (0.89–0.99)
RV ESV (mL)	0.0 ± 11.3	−22.1 to +54.6	1.000	0.90 (0.69–0.97)
RV EF (%)	−1.5 ± 10.0	−21.0 to +51.2	0.579	0.60 (−0.28–0.87)
Single-beat 30 fps	Bias	95% LOA	*p*	ICC, 95% CI
RV EDV (mL)	1.1 ± 14.3	−27.0 to +67.2	0.784	0.94 (0.80–0.98)
RV ESV (mL)	−2.7 ± 10.1	−22.5 to +54.1	0.332	0.88 (0.61–0.96)
EF (%)	1.0 ± 10.7	−19.9 to +49.7	0.740	0.27 (−1.53–0.77)
Multi-beat (4−6 beats)	Bias	95% LOA	*p*	ICC, 95% CI
RV EDV (mL)	1.1 ± 12.1	−22.6 to +56.3	0.729	0.96 (0.89–0.99)
RV ESV (mL)	1.9 ± 7.5	−12.8 to +32.7	0.371	0.96 (0.89–0.99)
RV EF (%)	−1.3 ± 5.5	−12.0 to +29.0	0.410	0.81 (0.43–0.94)

* statistically significant (*p* < 0.05). 3DE: three-dimensional echocardiography. RV EDV: right ventricular end-diastolic volume. RV ESV: right ventricular end-systolic volume. RV EF: right ventricular ejection fraction. LOA: limits of agreement. ICC: intraclass correlation.

**Table A3:** Comparison of different settings in 3DE single-beat acquisition with multi-beat acquisition (EDV 91.1 ± 24.4 mL; ESV 48.1 ± 16.4 mL) in patients (group 2). Mean volumes of single-beat acquisition are shown and compared to multi-beat results, using bias, paired Student *t*-test and Pearson’s correlation coefficient.

Single-beat, 20 fps	Mean	Bias	*p*	r
RV EDV (mL)	99.0 ± 29.1	−7.9 ± 17.1	0.026 *	0.81 (*p* < 0.001)
RV ESV (mL)	51.6 ± 17.0	−3.5 ± 2.2	0.134	0.77 (*p* < 0.001)
**Single-beat,** **25 fps**	**Mean**	**Bias**	***p***	**r**
RV EDV (mL)	91.1 ± 26.8	0.0 ± 21.8	1.000	0.64 (*p* < 0.001)
RV ESV (mL)	48.3 ± 19.9	−0.2 ± 13.4	0.942	0.75 (*p* < 0.001)
**Single-beat,** **30 fps**	**Mean**	**Bias**	***p***	**r**
RV EDV (mL)	85.3 ± 26.7	5.8 ± 20.2	0.155	0.69 (*p* < 0.001)
RV ESV (mL)	43.9 ± 17.1	4.3 ± 11.9	0.079	0.75 (*p* < 0.001)

* significant (*p* < 0.05). 3DE: three-dimensional echocardiography. RV EDV: right ventricular end-diastolic volume. RV ESV: right ventricular end-systolic volume. RV EF: right ventricular ejection fraction. *p*: paired student *t*-test. r: correlation coefficient.
